# On-Off Kinetics of Engagement of FNI Modules of Soluble Fibronectin by β-Strand Addition

**DOI:** 10.1371/journal.pone.0124941

**Published:** 2015-04-28

**Authors:** Wenjiang Ma, Hanqing Ma, Deane F. Mosher

**Affiliations:** Departments of Biomolecular Chemistry, University of Wisconsin-Madison, Madison, Wisconsin, United States of America; Russian Academy of Sciences, Institute for Biological Instrumentation, RUSSIAN FEDERATION

## Abstract

Intrinsically disordered sequences within bacterial adhesins bind to E-strands in the β-sheets of multiple FNI modules of fibronectin (FN) by anti-parallel β-strand addition, also called tandem β-zipper formation. The FUD segment of SfbI of *Streptococcus pyogenes* and Bbk32 segment of BBK32 of *Borrelia burgdorferi*, despite being imbedded in different adhesins from different bacteria, target the same FNI modules, ^2–5,8–9^FNI, in the N-terminal 70-kDa region (FN70K) of FN. To facilitate further comparisons, FUD, Bbk32, two other polypeptides based on SfbI that target ^1–5^FNI (HADD) and ^2–5^FNI (FRD), and mutant Bbk32 (ΔBbk32) were produced with fluorochromes placed just outside of the binding sequences. Unlabeled FUD competed ~1000-fold better for binding of labeled Bbk32 to FN than unlabeled Bbk32 competed for binding of labeled FUD to FN. Binding kinetics were determined by fluorescence polarization in a stopped-flow apparatus. On-rates for FUD, Bbk32, HADD, and FRD were similar, and all bound more rapidly to FN70K fragment than to full length FN. In stopped-flow displacement and size exclusion chromatographic assays, however, *k_off_* for FUD or HADD to FN70K or FN was considerably lower compared to *k_off_* of FRD or Bbk32. FUD and Bbk32 differ in the spacing between sequences that interact with ^3^FNI and ^4^FNI or with ^5^FNI and ^8^FNI. ΔBbk32, in which 2 residues were removed from Bbk32 to make the spacing more like FUD, had a *k_off_* intermediate between that of Bbk32 and FUD. These results indicate a “folding-after-binding” process after initial association of certain polypeptide sequences to FN that results in formation of a stable complex and is a function of number of FNI modules engaged by the polypeptide, spacing of engagement sites, and perhaps flexibility within the polypeptide-FN complex. We suggest that contributions of SfbI and BBK32 adhesins to bacterial pathogenicity may be determined in part by stability of adhesin-FN complexes.

## Introduction

**Fig 1:**
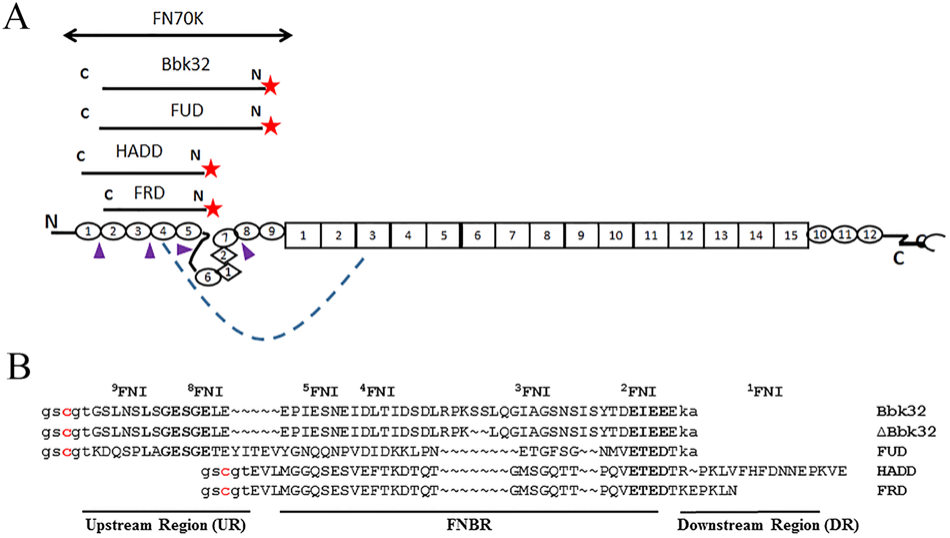
Plasma FN, FN70K, and polypeptides used in these studies. (A) Schematics of FN, FN70K, and polypeptides. Each plasma FN subunit consists of 12 FNI modules (ovals), 2 FNII modules (diamonds), and 15 FNIII modules (rectangles). The boundary of FN70K fragment is shown in reference to the FN domain structure. The polypeptides are aligned with the FN modules to which they bind. Their fluorochromes are indicated by asterisks. The flexible interfaces or connectors between FNI modules are indicated by arrowheads. The intra-molecular interaction between ^4^FNI and ^3^FNIII is indicated by dash line. (B) Sequences of FN-binding polypeptides. Gaps are introduced to align polypeptide sequences with the FNI modules to which the sequences are presumed to bind, as described in the text. The conserved LxGESGE and ExE motifs, which can be aligned to FNI modules with most confidence, are highlighted in bold. Lower case, residues introduced by cys-pET-Elmer. The cysteines marked in red were placed outside of the presumptive binding sequences to allow for specific labeling at a common site.

Fibronectin (FN) is a glycoprotein found in vertebrates as a disulfide-linked dimer. Each subunit of the FN dimer includes 12 type 1 (FNI), 2 type 2 (FNII) and 15–17 type 3 (FNIII) modules[1] (Fig 1A). It exists in a soluble, compact form in body fluids, which has been attributed to intra-molecular interactions within and between the two subunits[2–5]. FN becomes insoluble and extended when deposited into extracellular matrix in the process known as FN assembly[6].

FN is utilized by various bacteria for attachment and infection of host cells and tissues[7, 8]; in some cases FN binding results in invasion of cells[9–11]. Cell surface-attached proteins expressed by different bacteria, including FNBPA of *Staphylococcus aureus*, SfbI of *Streptococcus pyogenes*, FNZ of *Streptococcus dysgalactiae*, and BBK32 of *Borrelia burgdorferi*, contain intrinsically disordered regions that bind to FN through an unusual mechanism called tandem β-zipper formation[12–14]. Such proteins engage the FN70K domain comprising the N-terminal ^1-9^FNI and ^1-2^FNII modules of FN (Fig 1A) and form β–strands with the E-strands of sequential FNI modules in an interaction that can have low nM affinity[14, 15]. Although certain sequence motifs can be paired with specific FNI modules, isothermal titration calorimetric (ITC) studies of effects of mutations on a polypeptide based on SfbI failed to identify major “hot spots” and suggested that the binding energy is distributed across the interface[16]. Much more needs to be understood about tandem β-zipper formation and its role in bacterial pathogenesis.

Two of the most studied bacterial FN-binding proteins are SfbI and BBK32 of *S*. *pyogenes* and *B*. *burgdorferi*, respectively. SfbI contains an intrinsically disordered region consisting of five FN-binding repeats (FNBRs), each of which binds ^2-5^FNI, along with upstream and downstream flanking sequences that bind to ^8^FNI and ^1^FNI modules, respectively[12, 17, 18]. SfbI has been shown to mediate the bacterial invasion of cells by binding to FN[9, 19], a process that requires the upstream sequence[11]. The upstream or downstream sequence and its adjacent FNBR bind to FN with high affinity[12, 14, 17]. We call polypeptides based on the upstream sequence and adjacent FNBR and the downstream sequence and adjacent FNBR “FUD” and “HADD,” respectively, for “functional upstream domain” and “high affinity downstream domain”; the former binds ^2-5, 8-9^FNI, whereas the latter binds ^1-5^FNI[4, 16, 18]. BBK32 differs from SfbI in being linked to the bacterial cell surface by its N-terminus rather than the C-terminus[20] and having an intrinsically disordered FN-binding sequence that is non-repetitive[21]. The disordered sequence, however, when expressed as the “Bbk32” polypeptide engages the same extended binding site on FN as FUD with an affinity, as determined by ITC, that is ~4.5-fold less than the affinity of FUD[13].

It is remarkable that FUD and Bbk32, despite being imbedded in very different proteins of very different bacteria, bind to FN with a common tandem β-zipper formation mechanism. We hypothesized that a kinetic comparison of FUD and Bbk32 binding to FN would provide additional insights in sequence specificity and sequence-function relationship of bacterial adhesin binding to FN. To investigate such interactions by fluorescence polarization, we constructed FUD and Bbk32 with fluorochromes just N-terminal to the binding sequences (Fig 1B), We made similar constructs of HADD, FRD (for “functional repeat domain”) comprising a single FNBR[17], and an instructive mutant of Bbk32, ΔBbk32, which lacks the double serine (SS) in the linker between sequences binding to ^3^FNI and ^4^FNI (Fig 1B). When we compared the interactions of these polypeptides with FN or FN70K in stopped-flow experiments and complementary size exclusion chromatography experiments, we found striking differences in the stability of polypeptide-FN complexes that are of mechanistic and potential functional significance.

## Materials and Methods

### Plasma FN and FN70K fragment

Fig 1A depicts a schematic of plasma FN and FN70K fragment. FN was purified from a fibrinogen-rich plasma fraction by heat precipitation of fibrinogen (60°C, 5 min) followed by ion exchange chromatography[22]. Proteolytic FN70K fragment was prepared as described previously[23]. Protein concentrations were determined using extinction coefficients at 280 nm, which were calculated using the ProtParam tool from ExPASy. The molarity of FN or FN70K was calculated based on the mass of the monomer. A full-length FN monomer was assumed to have an average molecular mass of 250 kDa.

### Production and labeling of polypeptides

For FUD and HADD, the DNA sequence was cloned as described before[4, 18], and for FRD the DNA sequence was obtained by PCR strategy based on the sequence of HADD with the downstream sequence truncated. These DNA sequences were cloned into cys-pET-Elmer plasmid, which encodes a N-terminal polyhistidine tag that can be removed by thrombin cleavage, leaving a short N-terminal tail with a cysteine[24], after digestion of the plasmid and duplex cDNA. The coding sequence of Bbk32 was created by annealing and extending 5’-CCACTAGGTACCGGAAGTTTAAATTCCCTTAGCGGTGAAAGTGGTGAATTGGAGGAGCCTATTGAAAGTAATGAAATTGATCTTACTATAGATTCTGATTTAAGGCC-3’ and 5’-CTAGCTGCTAGCTTACTCTTCCTCTATTTCATCAGTGTATGAAATAGAGTTTGATCCTGCAATGCCTTGTAAGGAACTCTTTGGCCTTAAATCAGAATCTATAGTAAGATC -3’ followed by cloning into cys-pET-Elmer plasmid after digestion of the plasmid and duplex cDNA with *NheI* and *NcoI*. Construction of ΔBbk32 is similar to that of Bbk32 except that the nucleotides encoding the double serines (underlined in the 3’-primer) were deleted. Polypeptides were expressed in *E*. *coli* BL21 (DE3) as described previously for FUD in pET-Elmer[18]. After purification and proteolytic removal of the polyhistidine tag and in preparation for labeling, polypeptides were reduced by 5mM DTT. DTT was removed from polypeptide by gel filtration on Sephadex G-25 (GE Healthcare Life Science) immediately before labeling. The concentration of FUD was determined using extinction coefficient at 280 nm, which was calculated using the ProtParam tool from ExPASy and had been validated previously by amino acid analysis [18]. The concentrations of HADD, FRD, Bbk32, and ΔBbk32 were determined using the bicinchoninic acid (BCA) assay (Pierce) with FUD as the standard. The cysteine of the polypeptide was labeled by fluorochrome Alexa 488-maleimide (Life Technology) or maleimide-PEG2-biotin (Pierce) as per the manufacturer’s instructions. The Alexa fluorochrome labeled derivatives are hereafter called AF(polypeptide) and biotinylated polypeptides are called b-(polypeptide). Labeled polypeptide was purified by HiTrap Q column (GE Healthcare Life Science) and extensive dialysis. Mass spectrometric characterization by MALDI-TOF was performed to show that the expected product had been made.

### Enzyme-linked binding assays

Competitive binding assays were carried out as previously described[4, 18]. FUD or Bbk32, 1, 10, 100 or 1000 nM, was mixed with 0.5 nM b-Bbk32 or b-FUD, and the relative amount of b-Bbk32 or b-FUD binding to FN coated on 96-well plate was determined with HRP conjugated streptavidin (Costar 3590).

### Fluorescence polarization binding assay

Experiments were performed in 20mM Tris, 100mM NaCl, pH 7.4 buffer (TBS) with 0.1% bovine albumin on 96-well plate (Costar #3915) at 25°C by Tecan Genios Pro microplate reader with excitation 485nm and emission 535nm. The polarization value (P) was recorded. The dimensionless number P is expressed in millipolarization units throughout the paper. (1 Polarization Unit = 1000 mP Units). In the competitive binding assay, unlabeled FUD or Bbk32, 5, 10, 20, 50, 100, 200, 500 or 1000 nM, was added to FN-AFBbk32 or FN-AFFUD complex to displace AFBbk32 or AFFUD. Baseline polarization with AFBbk32 or AFFUD alone was subtracted from the polarization value of each experiment, and change in polarization was expressed as percentage of change in polarization of FN-AFFUD or FN-AFBbk32 complex in the absence of potential competitor.

### Stopped-flow fluorimetry for kinetic analysis

Measurements were in TBS with 100mM NaCl at 25°C. Reactants separated in two syringes were injected by SFA-20 rapid kinetics accessory (Hi-Tech Scientific, Salisbury, England) into the cuvette for mixing in QuantaMaster 300 spectrofluorimeter (Photon Technology International, Edison, New Jersey) with excitation and emission polarizers of 90 degree. The excitation and emission wavelength were 485nm and 535 nm respectively. Upon mixing the fluorescence intensity were recorded at a rate of 10 points/sec during the reaction procedure. In the absence of FN or FN70K, there was no change in fluorescence polarization for any of the AFpolypeptides.

In direct binding assays, AFFUD, AFHADD, AFFRD or AFBbk32, 10 nM, was mixed with a series of FN concentrations or with FN70K at 100 nM. AFΔBbk32 was studied only with FN at 100 nM. For each experiment, four or five curves were generated, and *k*_*obs*_ was calculated by

$$F(t)\;=\;F_{min}+(F_{max}-F_{min})e^{-k_{obs}t}$$ (1)

where F(t) is fluorescence intensity at time point t. F_max_ or F_min_ is maximum or minimum fluorescence intensity, and the rate constant k is *k*_*obs*_. For a given condition, the curves overlapped. Therefore, the *k*_*obs*_s were averaged for further analysis. To determine *k*_*on*_ and *k*_*off*_ of FN-AFpolypeptide, [FN] was plotted against *k*_*obs*_ and simulated by eq [2]:

$$k_{obs}\;=\;k_{on}{\left[FN\;or\;FN70K\right]}_{i}+\;k_{off}$$ (2)

*k*_*on*_ is the association rate constant, and *k*_*off*_ is the dissociation rate constant.

In displacement assays, unlabeled FUD, HADD, FRD, Bbk32, or ΔBbk32 in 10-fold molar excess of FN or FN70K was mixed with complex of AFpolypeptide and FN or FN70K. For ΔBbk32, only displacement from FN was measured. The *k*_*off*_ value is calculated by

$$F\left(t\right)\;=\;F_{min}+\left(F_{max}-F_{min}\right)\left(1-e^{-k_{off}t}\right)$$ (3)

in which *k*_*off*_ is the dissociation rate constant. The *k*_*on*_ of FN70K-AFpolypeptide was determined by Eq [2] based on [FN70K] and the *k*_*off*_ of FN70K-AFpolypeptide obtained from Eq [3].

### Size exclusion chromatography of FN-AFFUD or FN-AFBbk32 complex

To compare the *k*_*off*_ of FN-AFFUD and FN-AFBbk32 by a method that does not require addition of unlabeled ligand, 400 nM FN and 40 nM AFFUD or AFBbk32 were mixed together in TBS containing 100 mM NaCl and separated on a Superose 6, 10/300 size exclusion column (GE Healthcare Life Science) at a rate of 0.5 ml/min. Fractions of 0.5 ml were collected and assayed. FN was monitored by absorbance at 280 nm by NanoDrop 2000 UV-Vis Spectrophotometer (Thermo Scientific, Wilmington, DE) and AFpolypeptide by Tecan Genios Pro microplate reader with excitation 485 nm and emission 535 nm. FN, AFFUD or AFBbk32 was analyzed by itself as a control. The *k*_*off*_ of FN-AFFUD complex was estimated by estimating the lower limit of half-life (t_1/2_) of complex decay and the following equation:

$$t_{1/2}\;=\;\frac{0.693}{k_{off}}$$ (4)

## Results

### FUD and Bbk32 cross-competition for binding to FN is asymmetric

**Fig 2:**
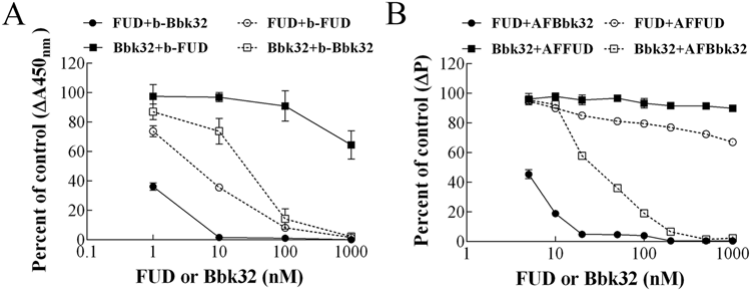
Cross-competition of binding to FN between FUD and Bbk32. (A) Competition for binding to coated FN. Increasing concentrations of FUD or Bbk32 were mixed with 0.5nM b-FUD or b-Bbk32 and incubated for 1 h in wells previously coated with 50 μl FN, 20nM. The amount of bound b-FUD or b-Bbk32 was determined based on turnover of substrate by alkaline phosphatase-avidin conjugate and expressed as percentage of ΔA450nm in the absence of any potential competitor. (B) Displacement from soluble FN. AFFUD or AFBbk32, 10 nM, was incubated with 100 nM FN for 30 min, after which increasing concentrations of unlabeled FUD or Bbk32 were added to the complexes. After 1 h, fluorescence polarization of each mix was recorded. Baseline polarization with AFBbk32 or AFFUD alone was subtracted from the polarization value, and change in polarization was expressed as percentage of change in polarization (ΔP) of FN-AFFUD or FN-AFBbk32 complex in the absence of potential competitor. Error bars represent mean ± SD of triplicate wells; for some points, error bars are smaller than the point symbol. The data in each panel are representative of three experiments.

As described in the Introduction, FUD and Bbk32 recognize a common elongated binding site comprising ^2-5^FNI and ^8-9^FNI of FN or its FN70K fragment[13, 17, 18]. By ITC, FUD or Bbk32 interacts with FN with a K_D_ of 26 or 121 nM[13]. The differences in affinity of FUD versus Bbk32, therefore, are ~4.5-fold. However, in pilot cross-competition experiments utilizing FUD or Bbk32 randomly biotinylated at amino groups, and an enzyme-linked assay of binding to adsorbed FN, unlabeled FUD effectively blocked binding of biotinylated FUD or Bbk32 whereas unlabeled Bbk32 only blocked binding of randomly biotinylated Bbk32 (results not shown). Since these results were obtained with heterogeneous probes, we made versions of FUD and Bbk32 in which a cysteine was introduced just N-terminal to the binding sequence, thus allowing targeted biotinylation (b-) or fluorescent labeling (AF) (Fig 1B). In an enzyme-linked assay for binding, after 1 h incubation of coated FN with mixtures of 0.5 nM b-FUD or b-Bbk32 and increasing concentrations of unlabeled FUD or Bbk32, FUD completely inhibited b-Bbk32 binding at a concentration as low as 10 nM, whereas more than 60% of b-FUD binding was present at a concentration of 1000 nM Bbk32 (Fig 2A). In self-competition assays performed the same way, unlabeled FUD or Bbk32 competed with b-FUD or b-Bbk32 with half maximal competition being achieved at ~10 nM for FUD and ~30 nM for Bbk32 (Fig 2A).

The huge asymmetry in cross-competition was at odds with self-competition and the ~4.5-fold differences in the K_D_ of the FN-FUD and FN-Bbk32 interactions as determined by ITC. To investigate if this asymmetry is due to a differential ability of FUD or Bbk32, once bound, to be displaced from FN, AFFUD or AFBbk32, 10 nM, was incubated with FN in solution for 30 min and then with unlabeled FUD or Bbk32 for an additional 1 h, measuring the proportion of bound AFpolypeptide by fluorescence polarization and expressing results compared to the change in polarization that was found in the absence of unlabeled FUD or Bbk32 (Fig 2B). Neither unlabeled FUD nor Bbk32 was effective at displacing AFFUD, although FUD was somewhat better than Bbk32, whereas unlabeled Bbk32 or FUD displaced AFBbk32 with dose responses that were similar to those found in the enzyme-linked assay (Fig 2B). We interpreted these experiments as indicating that bound Bbk32 is readily exchangeable with unbound Bbk32 or FUD whereas bound FUD is not. To follow this lead and investigate the sequence determinants for the difference in complex stabilities, we did additional studies with AFFUD and AFBbk32, and also AFHADD, AFFRD and AFΔBbk32. HADD and FRD recognize ^1-5^FNI or ^2-5^FN, respectively, and binding of constructs similar to HADD and FRD to FN or FN fragments has been studied by ITC and surface plasmon resonance[4, 16, 17]. ΔBbk32 contains a shorter linker sequence between the ^3^FNI- and ^4^FNI-binding regions of Bbk32 (Fig 1B).

### Binding of polypeptides to FN or FN70K exhibit different kinetics

**Fig 3:**
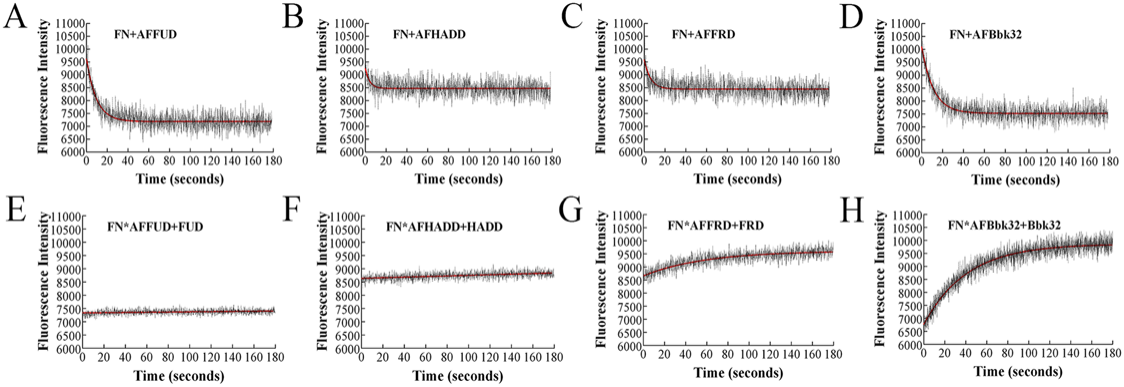
Binding of AFpolypeptide and FN as analyzed by stopped-flow fluorimetry. Upper set: AFFUD (A), AFHADD (B), AFFRD (C), or AFBbk32 (D), 10nM, was mixed with 300 nM FN. Fluorescence intensity was recorded with crossed polarizers in place. The data were fit into single exponential curves. The 300-nM curves shown here are representative of other curves for 100, 200, 400, and 500 nM FN and were analyzed together with the other curves to generate the plots in Fig 6. Lower set: AFFUD (E), AFHADD (F), AFFRD (G), or AFBbk32 (H), 10nM, was pre-mixed for 30 min with 100 nM FN, after which 1 μM unlabeled FUD, HADD, FRD, or Bbk32, respectively, was mixed with the complex in the stopped-flow apparatus to displace labeled polypeptide. Fluorescence intensity was recorded in the presence of crossed polarizers. The data were fit into single exponential curves.

Stopped-flow fluorimetry with crossed-polarizers was performed to study on-off kinetics of the FN-AFpolypeptide interactions. Upon mixing 10 nM AFFUD, AFHADD, AFFRD or AFBbk32 with increasing concentration (100, 200, 300, 400, or 500 nM) of FN, decreases of fluorescence intensity were observed (Fig 3). When crossed-polarizers were not in place, a small decrease in fluorescence intensity was found with AFFUD or AFBbk32 but not with HADD or FUD (data not shown). Therefore, the larger change in intensity of AFFUD (Fig 3A) or AFBbk32 (Fig 3D) compared to AFHADD (Fig 3B) or AFFRD (Fig 3C) is presumably due to the additive effects of fluorescence quenching and increased polarization for AFFUD or AFBbk32, whereas only increased polarization accounts for the lesser change in AFHADD or AFFRD. This explanation is compatible with the model described in Fig 1A in which the fluorochrome of AFHADD or AFFRD is positioned near the flexible linker region of ^5^FNI-^6^FNI whereas the fluorochrome in AFFUD or AFBbk32 is near the ^8-9^FNI/^1^FNIII junction. Curves for 10 nM AFpolypeptide binding to 300 nM FN are shown; these and the rest of the curves, not shown, were used to generate the linear regression plots and estimates of *k*_*on*_ and *k*_*off*_ in Table 1 as described below.

**Table 1:** Kinetics parameters of polypeptide-FN and polypeptide-FN70K interactions.

	*k*_*on*_ by multiple [FN] (Fig 5)(*M* ^-1^ ⋅ *s* ^-1^ × 10^-5^)	*k*_*off*_ by multiple [FN] (Fig 5) (*s* ^-1^ × 10^2^)	*k*_*on*_ by single [FN70K](*M* ^-1^ ⋅ *s* ^-1^ × 10^-5^)	*k*_*off*_ by displacement or chromatography (*s* ^-1^ × 10^2^)	K_D_ by on- and off-rate constants (nM)
**FUD-FN**	3.9 ± 0.2	0–0.57	—	< 0.1	< 2.6
**HADD-FN**	4.3 ± 0.3	0–2.5	—	< 0.1	< 2.3
**FRD-FN**	2.9 ± 0.6	2.7–3.9	—	2.1 ± 0.1	70
**Bbk32-FN**	1.8 ± 0.2	3.6–4.1	—	2.5 ± 0.03	140
**FUD-FN70K**	—	—	39± 1.8	< 0.1	< 0.26
**HADD-FN70K**	—	—	97± 1.5	< 0.1	< 0.10
**FRD-FN70K**	—	—	41 ± 3.4	1.3 ± 0.04	3.1
**Bbk32-FN70K**	—	—	24± 0.6	3.3 ± 0.04	14

To obtain *k*_*off*_ of FN-AFpolypeptide interaction by a different method, displacement assays were carried out in which 1 μM unlabeled FUD, HADD, FRD or Bbk32 was added into the complex of 10 nM AFFUD, AFHADD, AFFRD or AFBbk32 with 100 nM FN. Return of fluorescence intensity to that of the free AFpolypeptide was observed for FN-FRD (Fig 3G) and FN-Bbk32 (Fig 3H), whereas little change in intensity was detected for FN-FUD (Fig 3E) or FN-HADD (Fig 3F). Thus, for FN-AFFRD or FN-AFBbk32, the fluorescence intensity decrease in Fig 3C or Fig 3D nearly completely reversed (Fig 3G and 3H), whereas for FN-AFFUD or FN-AFHADD, the fluorescence intensity decrease in Fig 3A or Fig 3B reversed less than 10% over 180 sec (Fig 3E and 3F)

**Fig 4:**
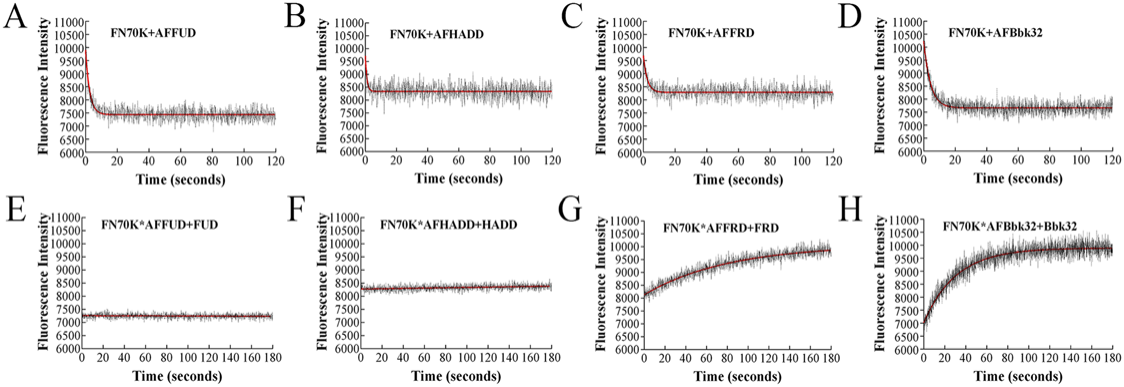
Binding of AFpolypeptide and FN70K as analyzed by stopped-flow fluorimetry. Upper set: AFFUD (A), AFHADD (B), AFFRD (C), or AFBbk32 (D), 10 nM, was mixed with 100 nM FN70K. Fluorescence intensity was recorded with crossed polarizers in place. Data were fit into single exponential curves, and observed rate constant of association reaction (*k*_*obs*_) was determined. Lower set: AFFUD (E), AFHADD (F), AFFRD (G), or AFBbk32 (H), 10nM, was pre-mixed for 30 min with 100nM FN70K, after which 1 μM unlabeled FUD, HADD, FRD, or Bbk32, respectively, was mixed with the complex in the stopped-flow apparatus to displace labeled polypeptide. Fluorescence intensity was recorded in the presence of crossed polarizers. The data were fit into single exponential curve.

Stopped-flow fluorimetry with crossed-polarizers was also performed for FN70K-AFpolypeptide interactions (Fig 4A–4D). The reaction was so rapid that the error of observed rate constants (*k*_*obs*_) with increasing concentration of FN70K would obscure any differences among *k*_*obs*_ at different FN70K concentrations. Thus, only a single concentration, 100 nM, of FN70K was tested. In the displacement assays, 1 μM unlabeled FUD, HADD, FRD or Bbk32 was added into the complex of 10 nM AFFUD, AFHADD, AFFRD or AFBbk32 with 100 nM FN70K (Fig 4E–4H). Similar to the results in Fig 3 for FN-AFpolypeptide, the fluorescence intensity decrease in Fig 4C or Fig 4D also reversed for FN-FRD (Fig 4G) and FN-Bbk32 (Fig 4H), whereas little reverse of fluorescence intensity was detected for FN-FUD (Fig 4E) or FN-HADD (Fig 4F).

**Fig 5:**
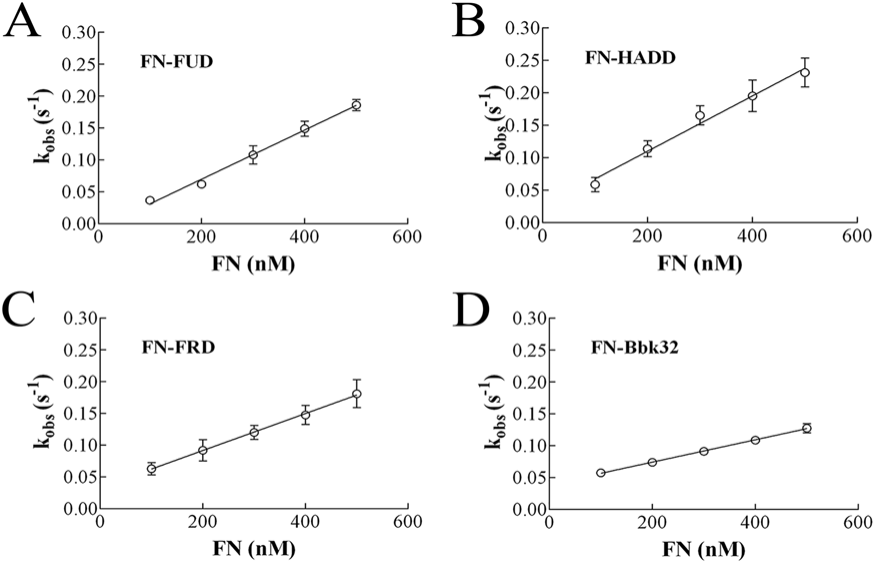
Determination of rate constants for FN-polypeptide interactions. Observed rate constant of association reaction (*k*_*obs*_) of AFFUD (A), AFHADD (B), AFFRD (C), or AFBbk32 (D) binding to each concentration of FN was determined in direct binding experiments. Plots of *k*_*obs*_ values versus concentration are shown based on data shown in Fig 3 and data not shown. *k*_*on*_ values from the slopes and *k*_*off*_ values estimated by the 95% confidence intervals of the y-intercepts are listed in Table 1.

Binding data were fit into a single exponential curve by Eq [1] and *k*_*obs*_ was calculated. Based on the calculated *k*_*obs*_ and concentration of FN ([FN]), a plot of *k*_*obs*_ versus [FN] was generated (Fig 5). Regression analysis indicated that *k*_*obs*_ of FN-AFpolypeptide versus [FN] was in a linear relationship. Association rate constants (*k*_*on*_) determined from Eq 2 are given in Table 1. Eq [2] allows the determination of dissociation rate constant (*k*_*off*_) of FN-AFpolypeptide interaction by extrapolation of the y-intercept, which are given in Table 1 as ranges falling within a 95% confidence interval for the extrapolations. *k*_*off*_ was also estimated from the displacement experiments. In accordance with the estimation of *k*_*off*_ values based on Fig 5, *k*_*off*_ of FN-FUD or FN-HADD was too low to be determined, and only the data of FN-FRD or FN-Bbk32 could be fitted to a single exponential curve (Eq [3]) allowing a calculation of *k*_*off*_ (Table 1). The *k*_*on*_ of FN70K-AFpolypeptide was calculated based on *k*_*obs*_, *k*_*off*_ and Eq [2]. For FN70K-FUD and FN70K-HADD, in calculating *k*_*on*_ the *k*_*off*_ was considered to be 0.

Inspection of Table 1 reveals that *k*_*on*_ of FN-AFpolypeptide interaction did not differ much among FN-AFFUD, FN-AFHADD, FN-AFFRD or FN-AFBbk32: 3.9, 4.3, 2.9, or 1.8 × 10^5^ M^-1^•s^-1^, respectively (Table 1). In contrast, the ranges of *k*_*off*_ of the FN-AFpolypeptide interaction were very different as estimated by Eq [2] or the displacement experiments. For FN-AFBbk32 or FN-FRD, *k*_*off*_ was 2.5 or 2.0 × 10^-2^ s^-1^ whereas for FUD or HADD, *k*_*off*_ was too low to be determined accurately. Kinetic parameters of the AFpolypeptide-FN70K interactions are also given in Table 1. These data indicate that although the *k*_*on*_ values of FN70K-AFpolypeptide interactions are 10- to 20-fold greater compared to the values of FN-AFpolypeptide, among different AFpolypeptides the rates are close. The higher *k*_*on*_ of FN70K-polypeptide interaction compared to FN-polypeptide has also been observed by surface plasmon resonance for a polypeptide similar to HADD[16] and attributed to the lack of the ^4^FNI-^3^FNIII intra-molecular interaction in FN70K[3, 4] (see Fig 1A). The *k*_*off*_ values of FN70K-AFpolypeptide interactions were similar to the values of FN-AFpolypeptide. For FN70K-AFBbk32 or FN70K-AFFRD, *k*_*off*_ was 3.3 or 1.3 × 10^-2^ s^-1^ (Table 1), whereas for FN70K-AFFUD or FN70K-AFHADD, *k*_*off*_ was too low to be determined. K_D_s of reactions calculated by $\frac{k_{off}}{k_{on}}$ are also given in Table 1.

### The FN-FUD complex is more stable than the FN-Bbk32 complex in size exclusion chromatography

**Fig 6:**
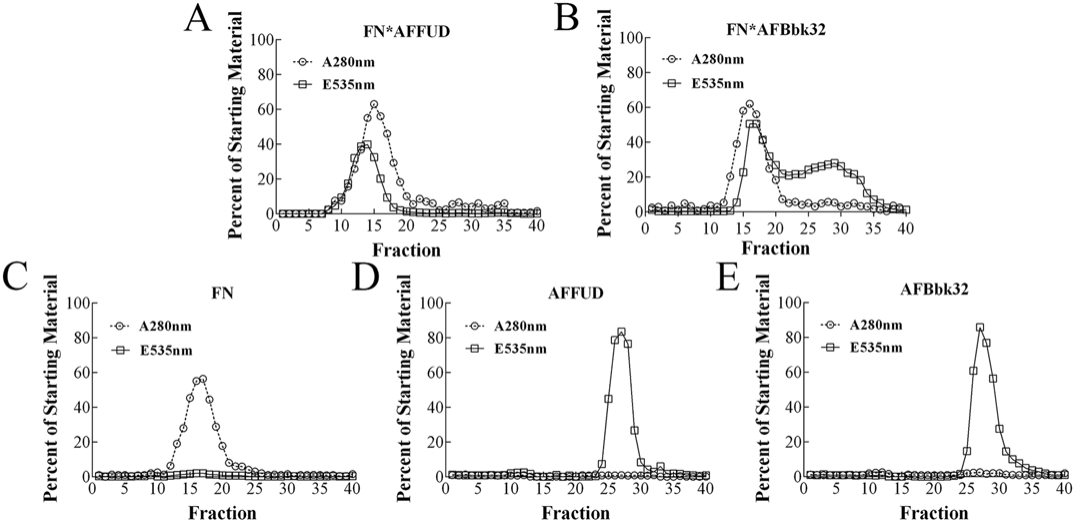
FPLC size exclusion of FN-AFFUD or FN-AFBbk32 complex. AFFUD (A) or AFBbk32 (B), 40nM, was mixed with 400nM FN for 30 min. The complex was loaded on a Superose 6 10/300 size-exclusion column and separated on FPLC. 400 nM FN (C), 40 nM AFFUD (D), or 40 nM AFBbk32 (E) alone was loaded on the same column. Absorbance at 280nm (A^280nm^), and emission at 535 nm upon excitation at 485 nm (E^535nm^) were measured to estimate, respectively, concentration of FN and AFpolypeptide and expressed relative to the values in starting material. Data are representative of three experiments.

To corroborate the estimates from stopped-flow fluorimetry of *k*_*off*_ for the FN-FUD or FN-Bbk32 interaction by a second method, size exclusion chromatography of the FN-AFFUD or FN-AFBbk32 complex was carried out. Almost 100% of AFFUD eluted towards the front of the FN peak (Fig 6A), indicating that AFFUD remained in complex with FN during passage through the column. In contrast, a major portion of AFBbk32 eluted continuously in fractions after the peak of FN, and only a lesser amount eluted in the trailing portion of the FN peak (Fig 6B). Elution patterns of FN alone (Fig 6C), AFFUD alone (Fig 6D), and AFBbk32 alone (Fig 6E) are also shown. FN eluted at 20 min. We estimate, therefore, that the half-life of FN-AFFUD is longer than 20 min and, according to Eq [4], the *k*_*off*_ of FN-AFFUD is < 10^-3^ s^-1^, which is more than 20-fold slower than that estimated for FN-AFBbk32 by Eq [2] or the displacement experiment (Table 1). Thus, the FN-FUD complex is more stable than FN-Bbk32 complex, which is consistent with the results in cross-competition (Fig 2) and stopped-flow fluorimetry (Table 1). The same experiment, carried out for FN70K-AFFUD, FN-AFHADD, or FN70K-AFHADD complex (data not shown), also demonstrated co-elution of fluorescence polypeptide and target with *k*_*off*_ values estimated as < 10^-3^s^-1^.

### ΔBbk32, a deletion mutant of Bbk32, forms a complex with FN that has higher stability than Bbk32

**Fig 7:**
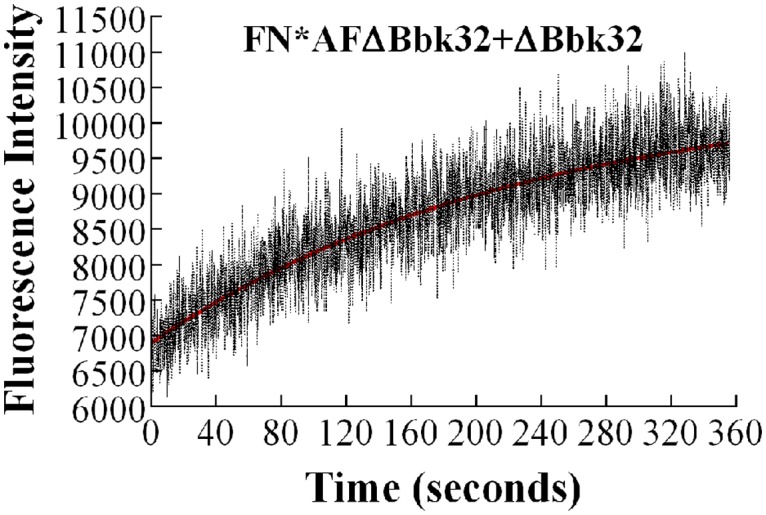
Displacement of AFΔBbk32 in FN-AFΔBbk32 complex. AFΔBbk32, 10nM, was pre-mixed for 30 min with 100nM FN, after which 1 μM unlabeled ΔBbk32 was mixed with the complex in the stopped-flow apparatus to displace AFΔBbk32. Fluorescence intensity was recorded with crossed polarizers in place.

The sequence alignment of FUD and Bbk32 shown in Fig 1B is based on previous literature describing NMR and crystallographic studies of binding of peptides taken from SfbI and BBK32 to pairs of FNI modules [13] and positions the peptide sequences underneath the cognate FNI modules. The more obviously similar sequences of FUD and Bbk32 are positioned below ^8^FNI and ^2^FNI at the N- and C- termini of the polypeptides, respectively, and the intervening, less obviously similar, binding sequences are positioned below ^5^FNI, ^4^FNI, and ^3^FNI. To achieve this alignment, a gap is introduced between the sequences in Bbk32 that bind to ^8^FNI and ^5^FNI, and an even larger gap is introduced between FUD sequences that bind to ^4^FNI and ^3^FNI. We hypothesized that length of intervening sequences linking binding sequences is important for complex stability and therefore studied a mutant Bbk32, ±Bbk32, with deletion of the double serine (SS) in the linker between sequences that bind to ^3^FNI and ^4^FNI (Fig 1B). Stopped-flow displacement experiments for FN-±Bbk32 complex yielded a *k*_*off*_ of 5.1 × 10^-3^ s^-1^ (Fig 7), which is 5-fold slower than the rate for FN-Bbk32 (Table 1). This observation indicates that mutated Bbk32 with the shortened sequence behaves more like FUD, including forming a more stable complex with FN.

## Discussion

Microbial adhesion to host tissues is an initial critical event in the pathogenesis of most infections[25]. Binding of intrinsically disordered region of bacterial surface proteins by β-zipper formation to tandem FNI modules that are unique to FN is a common mechanism of such adhesion[7], but how much specificity can be built into the mechanism is unknown. By comparing the kinetics of the binding between FN and polypeptides from BBK32 and different regions of SfbI, we provide new insights about both mechanism and specificity.

FUD, HADD, FRD, and Bbk32, all of which at a minimum interact with modules ^2-5^FNI, bound more quickly to FN70K than to FN. Beyond that, the polypeptides fell into two classes based on stopped-flow fluorescence polarization assays. For FUD and HADD, *k*_*off*_ s were >20-fold slower than for FRD and Bbk32 whereas *k*_*on*_ s of all the polypeptides were similar. The estimates of *k*_*off*_ s were corroborated by size exclusion chromatography. These data indicate that FUD or HADD forms stable complex with FN, whereas FRD or Bbk32 forms exchangeable complex. ΔBbk32, the deletion mutant of Bbk32, formed a more stable complex with FN than Bbk32 as indicated by a lower *k*_*off*_ of binding to FN.

**Fig 8:**
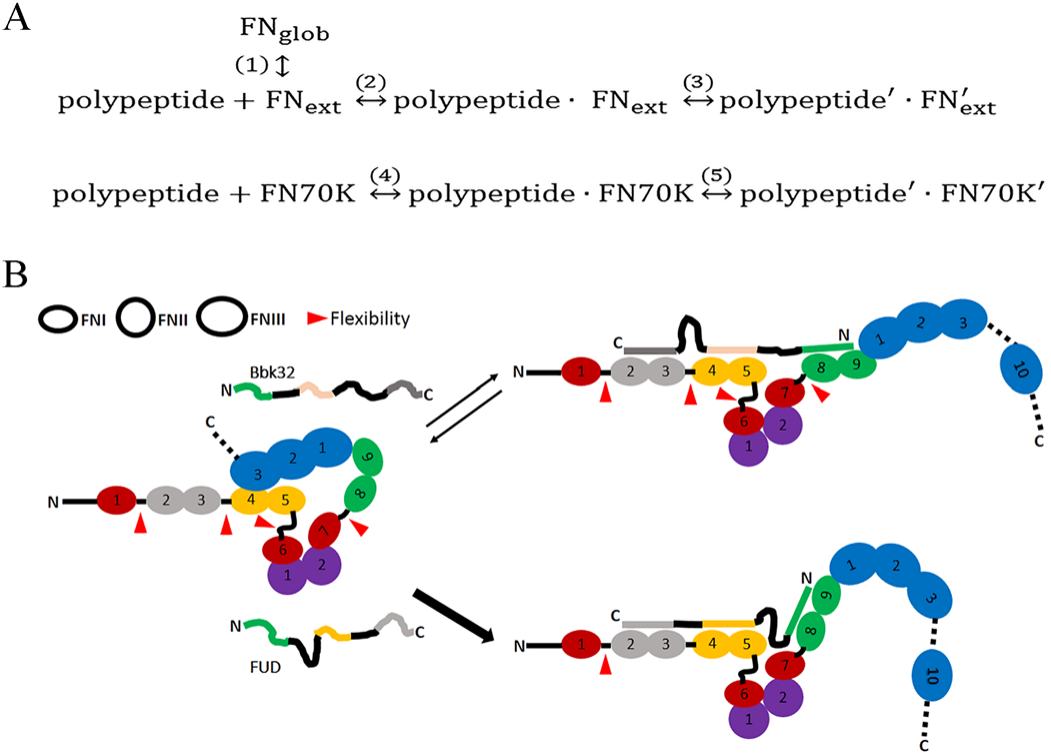
Schemas to rationalize differences among the polypeptide ligands. (A) FN is shown as being in a rapid equilibrium (reaction 1) between a globular conformation (FN_glob_) driven by the weak intra-subunit interaction between ^4^FNI and ^3^FNIII and a more extended conformation (FN_ext_) in which this interaction is lost. The binding site for all five polypeptides encompasses ^4^FNI, and thus binding is only to the more extended form of FN (reaction 2). After binding, we propose there are conformational changes (reaction 3) in both polypeptide (polypeptide’) and FN (FN’). In the case of FN70K, the binding site is immediately accessible in all molecules (reaction 4). The same conformational change (reaction 5) happens to polypeptide (polypeptide’) and FN70K (FN70K’) as occurs with polypeptide and FN. (B) Segments of FUD and Bbk32 and tandem FNI modules are colored-coded to indicate pairings that take place during complex formation. Both polypeptides are shown as displacing ^3^FNIII from ^4^FNI and causing extension of FN. In this hypothetical model, FUD is depicted as adapting differently to FN than Bbk32, thus forming a more stable complex in which FN has less module-module flexibility. The flexibility among FNI modules is indicated by arrowhead.

We propose a reaction scheme for the polypeptides binding to FN or FN70K in Fig 8 to rationalize these results. A weak intra-subunit interaction between ^4^FNI and ^3^FNIII of intact FN (dashed line in Fig 1A) has been demonstrated by NMR and complementary techniques[3–5]. Thus, we visualize FN as being in a rapid equilibrium between a more globular conformation (FN_glob_) driven by the ^4^FNI- ^3^FNIII interaction and a more extended conformation (FN_ext_) in which this interaction is lost (Fig 8A). The binding site for all four polypeptides encompasses ^4^FNI, and thus binding is only to the more extended form of FN. In the case of FN70K, binding to ^4^FNI is immediately accessible, thus accounting for the 9- to 23-fold faster *k*_*on*_ of the polypeptides interacting with FN70K compared to FN. To explain the differences in FUD and HADD compared to Bbk32 and FRD, we suggest that complexes of FN or FN70K and FUD or HADD undergo stabilizing conformational changes, denoted by “'“ in Fig 8A. Formation of stable FUD'-FN' or HADD'-FN' complex will favor the forward reactions of reactions 1 and 2, trapping the majority of FN in the extended form (FN_ext_). In contrast, Bbk32'-FN' or FRD'-FN' complex is less stable such that there is equilibration of all reactants, including FN_ext_ and FN_glob_, which results in exchangeable complexes and rapid displacement of bound polypeptide (Figs 3 and 4).

What determines complex stability? Comparing binding of FUD or HADD to FRD, inclusion of the upstream domain (as in FUD) or downstream domain (as in HADD) of SfbI protein greatly increased the stability of the complex. This result indicates that there is concerted or cooperative binding such that more interactions of the polypeptide with FN are preferred. In the case of FUD versus Bbk32, however, both polypeptides bind to the same number of tandem modules, ^2-3^FNI, ^4-5^FNI, and ^8-9^FNI, and short peptides from the two polypeptides bind to cognate tandem FN constructs with roughly the same affinity[13]. Thus, we hypothesize that the length and flexibility of intervening sequences within the polypeptides are important determinants of complex stability. In addition, we hypothesize that, although the structure and conformation of tandem FNI modules change very little upon binding to peptides in crystallographic studies[16, 26, 27], flexibility between individual or tandem modules of FN (arrowheads in Figs 1A and 8B) works hand-in-hand with flexibility in the polypeptides to allow the binding partners to adapt to one another. Sites of flexibility are depicted in Figs 1A and 8B. NMR studies of tandem ^1-2^FNI modules revealed no interactions, *i*.*e*., presence of flexibility, between ^1^FNI and ^2^FNI[14]. Crystal structures of un-ligated ^2-3^FNI demonstrated two configurations of the interface, only one of which is populated in the presence of peptide ligand[13, 16, 28]. There presumably is flexibility between ^3^FNI and ^4^FNI, although this issue has not been analyzed to our knowledge. ^4-5^FNI has been demonstrated by NMR to be locked in a single conformation[29]. The gelatin-binding region starting with ^6^FNI and ending with ^9^FNI adopts a non-linear conformation that is stabilized by multiple inter-modular interactions[30, 31]. The linker between ^5^FN and the gelatin-binding region is highly sensitive to proteolytic cleavage[1] and considered to be very flexible[32]. A recent structural analysis indicates flexibility in the linker of ^7^FNI and ^8^FNI by showing that the C-terminus of a type I collagen peptide binding to ^6-9^FNI leads to a 90° kink between ^7^FNI and ^8^FNI[33]. When FNI module pairs of a single FN subunit are able to engage different binding sites on a single polypeptide in a concerted manner, therefore, flexibility among FNI modules and within the polypeptide both would be constrained. For example, the FN-FUD and FN-HADD complexes would each constrain ^3-4^FNI flexibility with the FN-FUD interaction also constraining ^5^FNI-^8^FNI flexibility and the FN-HADD interaction also constraining ^1^FNI-^2^FNI flexibility. Thus, with FUD or HADD, three parts of the FN70K region would engage the polypeptide in concert so that the conformation of the complex is configured optimally in relation to FN and polypeptide, resulting in the low *k*_*off*_. Only two sites would need to be configured optimally for FRD, leading to a configuration that is less stable. Consistently, peptides that target ^1-3^FNI and ^4-5^FNI have been shown to bind less well when FN is subjected to mechanical strain[34]. This finding, plus a steered molecular dynamics simulation of effects of strain on a peptide-tandem FNI modules complex[34], supports the concept that consecutive FNI modules must be configured favorably relative to one another in order to engage bacterial polypeptides. Fig 8B depicts a speculative model of how binding of FUD-FN and Bbk32-FN may differ. The radically different spacing and sequences between binding sites to FN modules in Bbk32 compared to FUD may prevent the Bbk32-FN complex from adopting a favorable constrained conformation. This conjecture is supported by studies of the ΔBbk32 mutant in which the gap between sequences binding to ^2-3^FNI and ^4-5^FNI was altered to be more like FUD, resulting in more stable complex than with Bbk32. In addition, it was shown previously that FUD mutants with deletions as well as with block alanine substitutions of residues failed to compete with FUD for binding to FN in assays similar to that of Fig 2A[18], presumably because of failure of the mutants to form stable complexes with FN.

It is useful to consider the present findings in the context of current thinking about binding of intrinsically disordered sequences to structured proteins[35]. The two extremes of mechanisms proposed are the conformational selection and induced-fit models[36]. The first model posits a pre-existing transient structure in the intrinsically disordered sequence suitable for optimal binding whereas the second has the disordered sequence folding into the appropriate structure upon binding. Our data and the literature implicate features of both models. As described in Table 1, AFFUD, AFHADD, AFBbk32, and AFFRD, despite their sequence dissimilarity, bind to FN or FN70K with similar *k*_*on*_s, indicating that each assumes transient states in which one or more segments of the polypeptide exists in conformation(s) able to engage FNI modules. Previous studies indicated that the K_D_ of bacterial peptides from SfbI binding to ^1-2^FNI (0.4 μM) is lower than the K_D_s of peptides binding to ^2-3^FNI (3.6 μM) or ^4-5^FNI (113 μM)[14], consistent with finding that the sequence binding to ^1-2^FNI is predicted to have a higher propensity for β-strand formation[16], i.e., be in a pre-existing structure that is favorable for binding. The *k*_*off*_ s of the four polypeptides, however, are very different, indicating that the polypeptide and FN modules undergo distinct polypeptide-specific conformational change upon binding in an induced-fit process. Thus, we propose that in the binding reaction, initial association of polypeptide to FN or FN70K (reaction 2 or 4 in Fig 8A) is determined by the pre-existing, short-range transient structures in the polypeptide, and the stability of the complex (reaction 3 or 5 in Fig 8A) is determined by the induced-fit process. Such a hybrid mechanism also describes binding to FN of the R1R2 polypeptide based on the secreted SFS protein of *Streptococcus equi* subsp. *equi*[24]. R1R2 has two identical repeats that bind to ^8^FNI in a two-step process—rapid, readily reversible binding to ^8^FNI on one FN subunit and slower binding to ^8^FNI on the second FN subunit to form a much more stable complex.

We are struck that the unique kinetics of β-zipper formation are consistent with patterns of infection and dissemination. Infection with *S*. *pyogenes* involves long-term colonization of tissues such as the pharynx[37]; such persistence of the pathogen has been shown to be related to SfbI[38]. The low *k*_*off*_ s of FN-FUD and FN-HADD would allow SfbI and FN to form stable complexes and long-term adhesion to host cells. However, virulence of *S*. *pyogenes* is greater for SfbI-negative bacteria than the SfbI-expressing strain in mouse models in which the end point is dissemination of bacteria instead of colonization[39]. Lack of SfbI would result in failure to form stable complexes with FN and allow rapid dissemination of the bacteria to multiple tissues. It should be noted that although a single FNBR in the form of FRD did not form a stable complex with FN, there may be cooperative interactions between FUD, HADD, and intervening FNBRs that allow these complexes to be more stable[17]. A simplified picture of SfbI adhesion to FN could be as follows: the process is initiated with one FN molecule forming a stable complex with one FNBR plus the upstream or downstream sequence by concerted binding (resembling the FN-FUD or FN-HADD interaction), resulting in conformation changes of SfbI to allow adjacent FNBRs to recruit more FN molecules[17]. This hypothesis is consistent with the previous study showing that both FUD and HADD cause FN conformational extension with integrin binding site exposed[4], which presumably results in recruitment of integrins to trigger bacterial cell adhesion and internalization[9]. Unlike SfbI, BBK32 contains only one disordered FN binding region. Animal studies have demonstrated that rapid hematogenous dissemination of *B*. *burgdorferi*, a multistage process that includes tethering, dragging, stationary adhesion and extravasation, requires BBK32[40, 41]. This process fits well with the kinetic features of the FN-Bbk32 interaction; the high *k*_*on*_ would enable rapid initial attachment of bacteria cell to FN, and the relatively high *k*_*off*_ would allow separation of BBK32 from FN and dissemination of the pathogen. Speculations such as these must be evaluated alongside the fact that SfbI or BBK32 is not the only surface protein of the bacterial pathogen that binds to cell proteins. For example, RevA of *B*. *burgdorferi* also binds to fibronectin and may confer more stable adhesion than BBK32[42]. Nevertheless, our methods lend themselves to a workflow by which features that determine kinetics of the adhesin-FN interaction can be identified and engineered into mutant adhesins for functional studies in bacteria.

## References

[1] Pankov R, Yamada KM. Fibronectin at a glance. J Cell Sci. 2002;115(Pt 20):3861–3. Epub 2002/09/24. . 12244123 10.1242/jcs.00059

[2] Rocco M, Infusini E, Daga MG, Gogioso L, Cuniberti C. Models of fibronectin. EMBO J. 1987;6(8):2343–9. Epub 1987/08/01. 3665879 10.1002/j.1460-2075.1987.tb02510.x PMC553638

[3] Johnson KJ, Sage H, Briscoe G, Erickson HP. The compact conformation of fibronectin is determined by intramolecular ionic interactions. J Biol Chem. 1999;274(22):15473–9. Epub 1999/05/21. . 10336438 10.1074/jbc.274.22.15473

[4] Maurer LM, Ma W, Eickstaedt NL, Johnson IA, Tomasini-Johansson BR, Annis DS, et al Ligation of the Fibrin-binding Domain by beta-Strand Addition Is Sufficient for Expansion of Soluble Fibronectin. J Biol Chem. 2012;287(16):13303–12. Epub 2012/02/22. doi: M111.294041 [pii] 10.1074/jbc.M111.294041 22351755 PMC3339936

[5] Vakonakis I, Staunton D, Ellis IR, Sarkies P, Flanagan A, Schor AM, et al Motogenic sites in human fibronectin are masked by long range interactions. J Biol Chem. 2009;284(23):15668–75. Epub 2009/04/16. doi: M109.003673 [pii] 10.1074/jbc.M109.003673 19366708 PMC2708863

[6] Singh P, Carraher C, Schwarzbauer JE. Assembly of fibronectin extracellular matrix. Annu Rev Cell Dev Biol. 2010;26:397–419. Epub 2010/08/10. 10.1146/annurev-cellbio-100109-104020 . 20690820 PMC3628685

[7] Henderson B, Nair S, Pallas J, Williams MA. Fibronectin: a multidomain host adhesin targeted by bacterial fibronectin-binding proteins. FEMS Microbiol Rev. 2011;35(1):147–200. Epub 2010/08/11. doi: FMR243 [pii] 10.1111/j.1574-6976.2010.00243.x . 20695902

[8] Patti JM, Allen BL, McGavin MJ, Hook M. MSCRAMM-mediated adherence of microorganisms to host tissues. Annu Rev Microbiol. 1994;48:585–617. Epub 1994/01/01. 10.1146/annurev.mi.48.100194.003101 . 7826020

[9] Ozeri V, Rosenshine I, Mosher DF, Fassler R, Hanski E. Roles of integrins and fibronectin in the entry of Streptococcus pyogenes into cells via protein F1. Mol Microbiol. 1998;30(3):625–37. Epub 1998/11/21. . 9822827 10.1046/j.1365-2958.1998.01097.x

[10] Ozeri V, Rosenshine I, Ben-Ze'Ev A, Bokoch GM, Jou TS, Hanski E. De novo formation of focal complex-like structures in host cells by invading Streptococci. Mol Microbiol. 2001;41(3):561–73. . 11532125 10.1046/j.1365-2958.2001.02535.x

[11] Talay SR, Zock A, Rohde M, Molinari G, Oggioni M, Pozzi G, et al Co-operative binding of human fibronectin to Sfbl protein triggers streptococcal invasion into respiratory epithelial cells. Cell Microbiol. 2000;2(6):521–35. Epub 2001/02/24. doi: cmi76 [pii]. . 11207605 10.1046/j.1462-5822.2000.00076.x

[12] Schwarz-Linek U, Höök M, Potts JR. Fibronectin-binding proteins of gram-positive cocci. Microbes Infect. 2006;8(8):2291–8. 10.1016/j.micinf.2006.03.011 . 16782385

[13] Harris G, Ma W, Maurer LM, Potts JR, Mosher DF. Borrelia burgdorferi protein BBK32 binds to soluble fibronectin via the N-terminal 70-kDa region, causing fibronectin to undergo conformational extension. J Biol Chem. 2014;289(32):22490–9. 10.1074/jbc.M114.578419 24962582 PMC4139255

[14] Schwarz-Linek U, Werner JM, Pickford AR, Gurusiddappa S, Kim JH, Pilka ES, et al Pathogenic bacteria attach to human fibronectin through a tandem beta-zipper. Nature. 2003;423(6936):177–81. Epub 2003/05/09. doi: 10.1038/nature01589 . 12736686

[15] Matthews JM, Potts JR. The tandem β-zipper: modular binding of tandem domains and linear motifs. FEBS Lett. 2013;587(8):1164–71. 10.1016/j.febslet.2013.01.002 . 23333654

[16] Norris NC, Bingham RJ, Harris G, Speakman A, Jones RP, Leech A, et al Structural and functional analysis of the tandem beta-zipper interaction of a Streptococcal protein with human fibronectin. J Biol Chem. 2011;286(44):38311–20. Epub 2011/08/16. doi: M111.276592 [pii] 10.1074/jbc.M111.276592 21840989 PMC3207447

[17] Marjenberg ZR, Ellis IR, Hagan RM, Prabhakaran S, Hook M, Talay SR, et al Cooperative binding and activation of fibronectin by a bacterial surface protein. J Biol Chem. 2011;286(3):1884–94. Epub 2010/11/10. doi: M110.183053 [pii] 10.1074/jbc.M110.183053 21059652 PMC3023484

[18] Maurer LM, Tomasini-Johansson BR, Ma W, Annis DS, Eickstaedt NL, Ensenberger MG, et al Extended binding site on fibronectin for the functional upstream domain of protein F1 of Streptococcus pyogenes. J Biol Chem. 2010;285(52):41087–99. Epub 2010/10/16. doi: M110.153692 [pii] 10.1074/jbc.M110.153692 20947497 PMC3003407

[19] Molinari G, Talay SR, Valentin-Weigand P, Rohde M, Chhatwal GS. The fibronectin-binding protein of Streptococcus pyogenes, SfbI, is involved in the internalization of group A streptococci by epithelial cells. Infect Immun. 1997;65(4):1357–63. 9119474 10.1128/iai.65.4.1357-1363.1997 PMC175140

[20] Kim JH, Singvall J, Schwarz-Linek U, Johnson BJ, Potts JR, Hook M. BBK32, a fibronectin binding MSCRAMM from Borrelia burgdorferi, contains a disordered region that undergoes a conformational change on ligand binding. J Biol Chem. 2004;279(40):41706–14. Epub 2004/08/05. doi: doi: 10.1074/jbc.M401691200 M401691200 . 15292204 10.1074/jbc.M401691200

[21] Probert WS, Kim JH, Höök M, Johnson BJ. Mapping the ligand-binding region of Borrelia burgdorferi fibronectin-binding protein BBK32. Infect Immun. 2001;69(6):4129–33. 10.1128/IAI.69.6.4129-4133.2001 11349087 PMC98480

[22] Mosher DF, Johnson RB. In vitro formation of disulfide-bonded fibronectin multimers. J Biol Chem. 1983;258(10):6595–601. . 6133865

[23] McKeown-Longo PJ, Mosher DF. Interaction of the 70,000-mol-wt amino-terminal fragment of fibronectin with the matrix-assembly receptor of fibroblasts. J Cell Biol. 1985;100(2):364–74. Epub 1985/02/01. 3155749 10.1083/jcb.100.2.364 PMC2113439

[24] Ma W, Ma H, Fogerty FJ, Mosher DF. Bivalent ligation of the collagen-binding modules of fibronectin by SFS, a non-anchored bacterial protein of Streptococcus equi. The Journal of biological chemistry. 2014 10.1074/jbc.M114.612259 . 25525266 PMC4335226

[25] Pizarro-Cerdá J, Cossart P. Bacterial adhesion and entry into host cells. Cell. 2006;124(4):715–27. 10.1016/j.cell.2006.02.012 . 16497583

[26] Bingham RJ, Rudino-Pinera E, Meenan NA, Schwarz-Linek U, Turkenburg JP, Hook M, et al Crystal structures of fibronectin-binding sites from Staphylococcus aureus FnBPA in complex with fibronectin domains. Proc Natl Acad Sci U S A. 2008;105(34):12254–8. Epub 2008/08/21. doi: 0803556105 [pii] 10.1073/pnas.0803556105 18713862 PMC2518095

[27] Erat MC, Slatter DA, Lowe ED, Millard CJ, Farndale RW, Campbell ID, et al Identification and structural analysis of type I collagen sites in complex with fibronectin fragments. Proc Natl Acad Sci U S A. 2009;106(11):4195–200. Epub 2009/03/03. doi: 0812516106 [pii] 10.1073/pnas.0812516106 19251642 PMC2649207

[28] Rudino-Pinera E, Ravelli RB, Sheldrick GM, Nanao MH, Korostelev VV, Werner JM, et al The solution and crystal structures of a module pair from the Staphylococcus aureus-binding site of human fibronectin—a tale with a twist. J Mol Biol. 2007;368(3):833–44. Epub 2007/03/21. doi: S0022-2836(07)00253-7 [pii] 10.1016/j.jmb.2007.02.061 . 17368672

[29] Baron M, Norman D, Willis A, Campbell ID. Structure of the fibronectin type 1 module. Nature. 1990;345(6276):642–6. 10.1038/345642a0 . 2112232

[30] Erat MC, Schwarz-Linek U, Pickford AR, Farndale RW, Campbell ID, Vakonakis I. Implications for collagen binding from the crystallographic structure of fibronectin 6FnI1-2FnII7FnI. J Biol Chem. 2010;285(44):33764–70. Epub 2010/08/27. doi: M110.139394 [pii] 10.1074/jbc.M110.139394 20739283 PMC2962475

[31] Graille M, Pagano M, Rose T, Ravaux MR, van Tilbeurgh H. Zinc induces structural reorganization of gelatin binding domain from human fibronectin and affects collagen binding. Structure. 2010;18(6):710–8. Epub 2010/06/15. doi: S0969-2126(10)00161-9 [pii] 10.1016/j.str.2010.03.012 . 20541508

[32] Atkin KE, Brentnall AS, Harris G, Bingham RJ, Erat MC, Millard CJ, et al The streptococcal binding site in the gelatin-binding domain of fibronectin is consistent with a non-linear arrangement of modules. J Biol Chem. 2010;285(47):36977–83. Epub 2010/09/17. doi: M110.156935 [pii] 10.1074/jbc.M110.156935 20843804 PMC2978626

[33] Erat MC, Sladek B, Campbell ID, Vakonakis I. Structural analysis of collagen type I interactions with human fibronectin reveals a cooperative binding mode. J Biol Chem. 2013;288(24):17441–50. 10.1074/jbc.M113.469841 23653354 PMC3682544

[34] Chabria M, Hertig S, Smith ML, Vogel V. Stretching fibronectin fibres disrupts binding of bacterial adhesins by physically destroying an epitope. Nat Commun. 2010;1:135 10.1038/ncomms1135 21139580 PMC3105298

[35] Uversky VN. Multitude of binding modes attainable by intrinsically disordered proteins: a portrait gallery of disorder-based complexes. Chem Soc Rev. 2011;40(3):1623–34. 10.1039/c0cs00057d . 21049125

[36] Kiefhaber T, Bachmann A, Jensen KS. Dynamics and mechanisms of coupled protein folding and binding reactions. Curr Opin Struct Biol. 2012;22(1):21–9. 10.1016/j.sbi.2011.09.010 . 22129832

[37] Moschioni M, Pansegrau W, Barocchi MA. Adhesion determinants of the Streptococcus species. Microb Biotechnol. 2010;3(4):370–88. 10.1111/j.1751-7915.2009.00138.x 21255337 PMC3815805

[38] Neeman R, Keller N, Barzilai A, Korenman Z, Sela S. Prevalence of internalisation-associated gene, prtF1, among persisting group-A streptococcus strains isolated from asymptomatic carriers. Lancet. 1998;352(9145):1974–7. 10.1016/S0140-6736(97)12452-7 . 9872247

[39] Nyberg P, Sakai T, Cho KH, Caparon MG, Fässler R, Björck L. Interactions with fibronectin attenuate the virulence of Streptococcus pyogenes. EMBO J. 2004;23(10):2166–74. 10.1038/sj.emboj.7600214 15103329 PMC424380

[40] Hyde JA, Weening EH, Chang M, Trzeciakowski JP, Höök M, Cirillo JD, et al Bioluminescent imaging of Borrelia burgdorferi in vivo demonstrates that the fibronectin-binding protein BBK32 is required for optimal infectivity. Mol Microbiol. 2011;82(1):99–113. 10.1111/j.1365-2958.2011.07801.x 21854463 PMC3183165

[41] Norman MU, Moriarty TJ, Dresser AR, Millen B, Kubes P, Chaconas G. Molecular mechanisms involved in vascular interactions of the Lyme disease pathogen in a living host. PLoS Pathog. 2008;4(10):e1000169 10.1371/journal.ppat.1000169 18833295 PMC2542414

[42] Brissette CA, Bykowski T, Cooley AE, Bowman A, Stevenson B. Borrelia burgdorferi RevA antigen binds host fibronectin. Infection and immunity. 2009;77(7):2802–12. 10.1128/IAI.00227-09 19398540 PMC2708576

